# Involuntary motor responses are elicited both by rare sounds and rare pitch changes

**DOI:** 10.1038/s41598-024-70776-x

**Published:** 2024-08-30

**Authors:** Simily Sabu, Fabrice B. R. Parmentier, János Horváth

**Affiliations:** 1 Institute of Cognitive Neuroscience and Psychology, HUN-REN Research Centre for Natural Sciences, P.O.B. 286, Budapest, 1519 Hungary; 2 Department of Psychology and Research Institute of Health Sciences (IdISBa), University of the Balearic Islands, Ctra. De Valldemossa, Km 7.5, Palma de Mallorca, Balearic Islands Spain; 3 School of Psychological Science, University of Western Australia, Perth, Australia; 4 Institute of Psychology, Károli Gáspár University of the Reformed Church in Hungary, Budapest, Hungary

**Keywords:** Human behaviour, Attention, Motor control

## Abstract

Unpredictable deviations from an otherwise regular auditory sequence, as well as rare sounds following a period of silence, are detected automatically. Recent evidence suggests that the latter also elicit quick involuntary modulations of ongoing motor activity emerging as early as 100 ms following sound onset, which was attributed to supramodal processing. We explored such force modulations for both rare and deviant sounds. Participants (N = 29) pinched a force sensitive device and maintained a force of 1–2 N for periods of 1 min. Task-irrelevant tones were presented under two conditions. In the *Rare condition*, 4000 Hz tones were presented every 8-to-16 s. In the *Roving condition*, 4000 Hz and 2996 Hz tones were presented at rate of 1 s, with infrequent (*p* = 1/12) frequency changes. In the Rare condition, transient force modulations were observed with a significant increase at ~ 234 ms, and a decrease at ~ 350 ms. In the Roving condition with low frequency deviant tones, an increase in force was observed at ~ 277 ms followed by a decrease at ~ 413 ms. No significant modulations were observed during perception of high frequency deviants. These results suggest that both rare silence-breaking sounds and low-pitched deviants evoke automatic fluctuations of motor responses, which opens up the possibility that these force modulations are triggered by stimulus-specific change-detection processes.

## Introduction

Human sensory and cognitive systems possess a remarkable ability to detect changes in one’s stimulus environment. This not only allows us to streamline the processing of sensory information and guide stimulus selection by triggering attentional change, but it also provides an opportunity to respond to these changes in an adaptive manner. In the auditory domain, the ability to detect sensory change is supported by several distinct mechanisms, which can be selectively engaged in different stimulation contexts. A set of recent studies^1,2^ found that stimuli presented after longer periods of silence not only capture attention, but also induce rapid, characteristic changes in continuous motor output. These motor changes, which occur as early as 100 ms after the sound onset, may prime quick and adaptive responses to salient stimuli. Auditory change, however, is also automatically detected in environments which feature constant, regular auditory stimulation: it is well known^3–5^ that sound events violating such regularities are automatically detected, which may trigger changes in attention. The goal of the present study was to investigate whether such regularity violations also resulted in motor responses similar to those triggered by sounds presented after longer periods of silence.

Adaptive behavior requires one to become aware of changes in one’s (sensory) environment. In the auditory domain, *rare stimuli* disrupting an auditory environment that is seemingly devoid of stimulation are detected automatically, i.e., when longer periods of silences are interrupted by stimuli occurring at unpredictable times or when a *deviant stimulus* that is distinguishable by its various features like frequency, loudness, temporal characteristics etc., stands out from a continuous stream of otherwise similar auditory stimuli^5–7^. An example of the former is a sudden noise of a breaking tree branch in the dead of night on a camping trip, and an example of the latter would be a change in the tire noise produced by one’s car during driving. In either of these cases, it should be noted that change detection only constitutes the initial step of a process resulting in an attentional shift toward the unexpected sound and the reappraisal of one’s actions to achieve the appropriate behavioral outcome^8^—be it suddenly moving away from the falling tree or stopping to check for a flat tire.

These initial processing steps contributing to bottom-up attention change are reflected by several event-related potential (ERP) waveforms: stimuli presented after longer periods of stimulation absence elicit the so-called vertex response^6,9^, a non-stimulus-specific ERP waveform. Infrequent sounds presented in the context of a regular sound sequence which deviate from this regularity (e.g. a high-pitched tone following several low-pitched tones) elicit the Mismatch Negativity (MMN), a distinct negative deflection that occurs around 150–250 ms after the occurrence of the deviant stimulus^10^. MMN is widely accepted as a neurophysiological marker for change detection, interpreted as a reflection of a process related to the mismatch between a sensory prediction based on the preceding regular stimulus sequence and the unexpected stimulus^3,11–14^. MMN-related processes may trigger an involuntary attentional shift to the deviant stimulus which may potentially hold relevant information, thus compelling the brain to prioritize its processing^15–20^. Alternative accounts provide evidence for stimulus-specific neuronal adaptation within the auditory cortex (reflected mainly by the adaptation of the auditory N1 ERP component), suggesting decreased neural responsiveness to the repeated standard stimuli, thus engaging previously unengaged neuronal elements for processing the deviant stimulus^21–26^. Recent perspectives on MMN and change detection incorporate both accounts, suggesting a hierarchical organization of the processing of regularity violations and neuronal adaptation^7,14,27–29^. Importantly, whereas the auditory vertex response is mainly observable for relatively long (longer than about 3–4 s^1,6^) inter-stimulus intervals, MMN and ERP components reflecting stimulus-specific adaptation are readily elicited when sounds follow each other at short (e.g. 200 ms^30,31^) intervals.

While such distinct cortical markers account for sensory and attentional engagement during change detection, it remains unclear how the motor system is affected during the same, especially considering that it often leads to a behavioral output, as mentioned in the beginning. There is some evidence showing how a predictable auditory change can elicit motor responses that are *voluntary* in nature, like the clapping of hands or the rhythmic tapping of feet when your playlist plays your favorite music. Studies have demonstrated that predictable auditory events can induce motor preparedness in individuals, even in the absence of an intention to move^32^. For example, Kilintari and colleagues^33^, showed that rare task-irrelevant auditory changes within long inter-target silences reduced the participant’s task-related movement onset times in a complex visuomotor task. While these are relevant in understanding the complexity of sensory-motor integration during change detection, it should be noted that motor responses to sudden changes are often *involuntary,* such as startle reflexes, orienting responses, or fight-or-flight responses. Such motor responses may unravel even when one is not prepared to act or anticipates an upcoming deviant sound^34^. Evidence indicates that a loud, startling sound affects ongoing motor control by yielding the early release of prepared actions^35^, and the effects of startling auditory stimuli on the motor cortex have also been well-documented^36,37^. In certain situations, however, unexpected sounds are not startling but may nevertheless call for a change in actions. For example, the hissing sound of a snake or the sound of a fire crackling in the room next door are auditory events that are faint, but if ignored, could have dire consequences. Relatively little is known about the mechanisms through which such unexpected sounds impact on the motor system.

Novembre and colleagues^1^ recently proposed that detection of salient sub-startle stimuli modulates ongoing motor activity. Asking participants to perform an isometric force task while exposed to unexpected auditory and electrical stimuli of varying intensity, they observed that such stimuli modulated the force produced by participants. This modulation took the form of three peaks, with a decrease in force as early as ~ 100 ms post stimulus-onset, an increase around 250 ms, and a further increase at around 350 ms. The magnitude of this modulation correlated with the amplitude of the EEG vertex response to these stimuli, thereby suggesting tight cortico-muscular coupling between sensory processing and action control. Such notion is also illustrated by the dynamic link observed between salient changes within a complex auditory stream and the involuntary motor responses elicited by these changes^34^. These results, as well as follow-up work from Novembre and colleagues^2,38^ makes a strong case for the audio-motor integration mechanism. Of interest, several studies have suggested that one important consequence of the detection of unexpected events on motor control is a transient and general inhibition of motor actions^8,39–44^. For example, Wessel and Aron^45^ demonstrated a temporary reduction in corticospinal excitability of the motor cortex at around 150 ms following the presentation of a novel sound. Recent work extended this conclusion to oculomotor behavior by showing that deviant sounds lead to an increase in fixation durations in a reading task, which appears to emanate from the perturbation of the planning of the next saccade^46,47^. The same effect was observed in reading and scanning tasks, further ruling out the possible role of lexical processes and landing the locus of the oculomotor perturbation on a global suppression of eye-movement control^48^.

Our study aims to further explore how unpredictable auditory changes elicit involuntary motor responses. Specifically, we investigated whether ongoing motor performance is affected by the presentation of infrequent auditory stimuli amid longer periods of silence (rare stimuli) – following up on the results of Novembre and colleagues^1^, and auditory stimuli breaking the regularity of a relatively fast-paced auditory stream (deviant stimuli). Participants performed an isometric force task in which they were required to produce continuous force within a range of 1–2 N while presented with blocks of rare or deviant tones in an interwoven design. Because previous studies connected involuntary motor fluctuations elicited by salient sounds to the vertex response, the elicitation of similar fluctuations in other auditory contexts known to elicit involuntary attention change would demonstrate that such motor modulations may not be exclusively related to the vertex response, but may also be triggered by other auditory change detection mechanisms.

## Materials and methods

### Overview of the experiments

The first experiment (described in detail in the Supplementary Material), hereafter referred to as the pilot experiment, provided force data that was contaminated by low-amplitude transient artifacts related to the operation of the computer’s sound card (a 30-ms-long spike occurring every 500 ms following the start of the sound sequence). Although contamination from these artifacts could be separated from stimulus-locked motor activity, we nonetheless decided to use the data from the pilot experiment for exploration and specification of an optimal processing and analysis pipeline only. The main focus of the exploration was the selection of the intervals of interest (see Supplementary Material). After the modification of the experimental setup to prevent the occurrence of the artifact, the second (main) experiment was administered. Data from this experiment was analyzed using the processing and analysis specified in the pilot experiment. Note that the analysis of the data from the main experiment took place only *after* the processing pipeline was specified—that is, the processing pipeline was specified without “peeking” at the second dataset. This approach prevented the inflation of the alpha-level that occurs when the same dataset is used both for the selection of the analyses and hypothesis testing (often referred to as “double dipping”^49,50^), and provided maximal statistical power to detect the observed effects in the second (main) experiment reported below.

### Participants

A total of 30 healthy human participants (17 females, mean ± SD age = 22.9 ± 2.50 years, range 19–28 years, 2 left-handed) participated in the experiment. The sample size was decided by initial analyses of the pilot experiment’s results in intervals selected based on the initial visual inspection of the group-average force signals. To provide a statistical power of 0.8 or above for the detection of force modulations within these intervals, a minimum sample size of 16 participants was needed. A sensitivity analysis based on the optimized processing pipeline is presented in the Statistical Analysis section below. One participant’s data was excluded based on the outlier rejections (see below); thus, data from 29 participants were included in the final analysis (17 females, mean ± SD age = 22.72 ± 2.35 years, range 19–28 years, 2 left-handed). All participants gave informed consent in written form prior to the experiment. The experiments were conducted in accordance with the Declaration of Helsinki. The project was approved by the United Ethical Review Committee for Research in Psychology (Hungary).

### Apparatus and stimuli

A single-zone Force Sensitive Resistor (FSR, FSR400 short, Interlink Electronics, 0.3 mm thick with an active area of 5.1 mm diameter) glued to a thin plastic sheet was used to measure the force applied by the participants. The FSR signal was recorded by a Synamp2 EEG amplifier (Compumedics Neuroscan, Victoria, Australia) with a sampling rate of 1000 Hz (online low pass filtered at 200 Hz). The voltage signal was transformed to force by an exponential transformation. The auditory stimuli were presented through HD-600 headphones (Sennheiser, Wedemark, Germany). Tone duration was 50 ms including 5–5 ms linear rise and fall times. The tones were presented with 50 dB above the individual’s 75% hearing threshold for the 4000 Hz tone determined by using the Single Interval Adjustment Matrix procedure^51,52^. The tone frequencies used in the different experimental conditions (see below) were 4000 Hz and 2996 Hz (i.e., five semitones lower than 4000 Hz) to match the stimulation used by Novembre and colleagues^1^. The experiment was run in GNU Octave^53^, using the Psychophysics Toolbox^54–56^ on a personal computer with a Linux operating system.

### Task and procedure

Participants were comfortably seated in an armchair in front of a computer screen, in a sound attenuated room. Using their dominant hand, participants held the FSR between the thumb and the index finger in a “thumb above” position (see Fig. S1, top left), while comfortably resting their forearm on the arm of the chair, or in their lap. They were required to pinch the FSR and maintain a constant force within the 1–2 N range. At the beginning of the experiment – in the familiarization phase – continuous visual feedback was provided by showing participants the FSR signal using the EEG recording software’s continuous display. After exploring the correspondence between the applied force and the FSR signal, participants were instructed to find a hand position that allowed the maintenance of a steady force level. Following the familiarization phase, the screen was turned off. In each experimental block, before starting the auditory stimulation, the experimenter provided continuous verbal feedback on the current level of force through an intercom. Once participants were able to maintain their force response within the target range, the auditory stimulation was initiated, which lasted for 60 s.

**Fig. 1:**
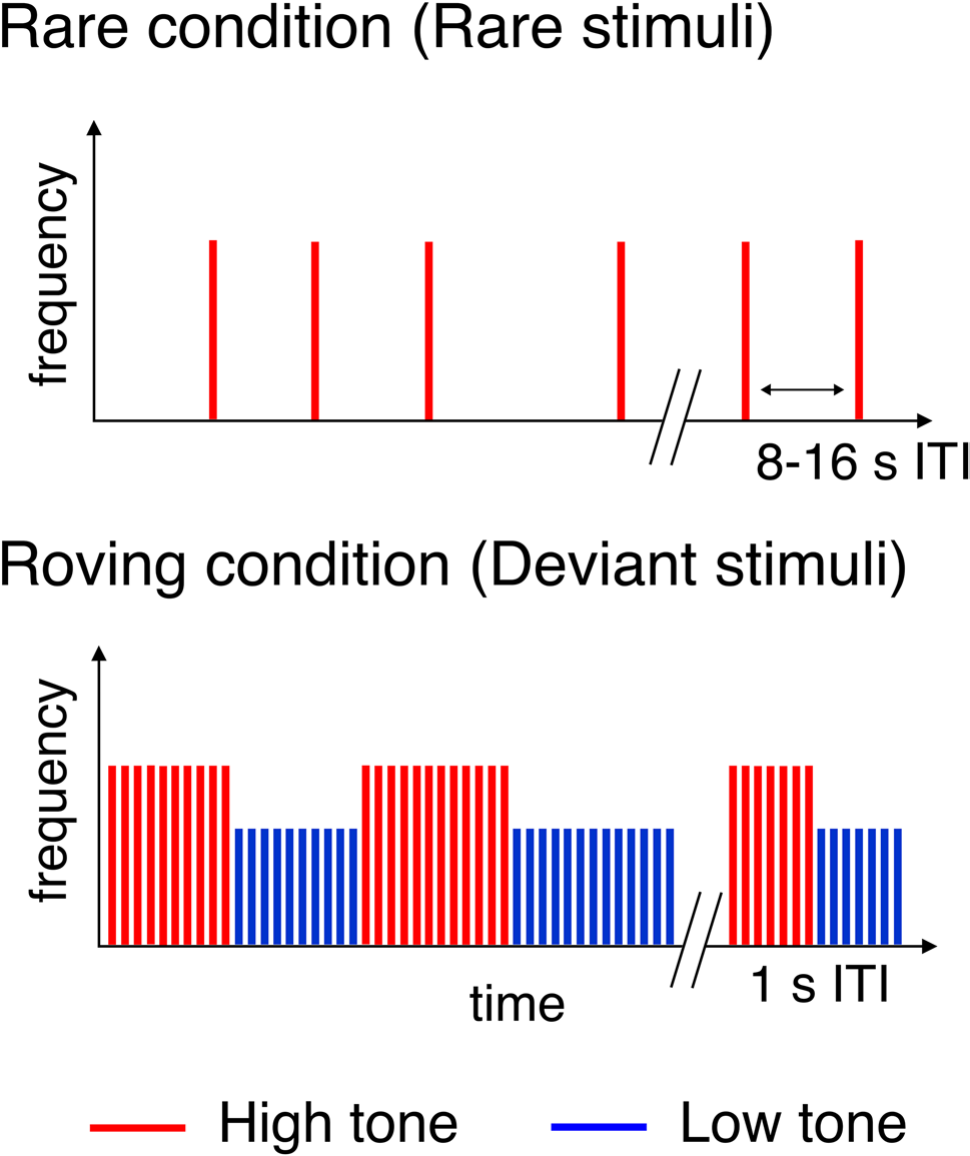
Experimental paradigm. Two experimental conditions were administered; the top panel illustrates the Rare condition consisting of rare high tones (4000 Hz) presented with an 8–16 s onset-to-onset interval. The bottom panel illustrates the Roving condition consisting of high (4000 Hz) and low (2996 Hz) tones presented with a 1 s onset-to-onset interval. In this condition, the same frequency tones were presented as microsequences that were at least 5 stimuli long, followed by a microsequence of the other tone. The frequency change acted as a deviant stimulus in this condition.

In the *Rare condition,* 4000 Hz pure tones were presented with 8–16 s (randomly sampled from a uniform distribution) onset-to-onset intervals, thus acting as *rare* tones occurring in-between periods of silences (see Fig. 1, top). In the *Roving* condition, high (4000 Hz) and low (2996 Hz) sinusoid tones were presented with 1 s onset-to-onset interval. The sequence consisted of micro-sequences of 5 or more tones of the same frequency (e.g., high tone), interleaved with micro-sequences of the other tone (e.g., low tone) to form a sequence of tones with rare changes of frequency (e.g., -HHHHHHHLLLLLL-). Five such changes (i.e., six homogeneous micro-sequences) were randomly placed in each block of 60 stimuli (Fig. 1, bottom), resulting in five changes per minute. Hereafter, frequency change following a homogeneous micro-sequence will be referred to as a *low* or *high deviant* depending on the frequency of the deviating tone*.* The Rare (Ra) and Roving (Ro) blocks were presented in an interwoven order as Ro-Ra-Ro-Ro-Ra-Ro-Ro-Ra-Ro-…-Ro-Ra-Ro. In total, there were 10 Rare blocks and 20 Roving blocks per participant.

### Data processing

Data was processed and analyzed using MATLAB (R2017b, The MathWorks, Inc., Natick, Massachusetts, United States), except for the power calculations which were conducted in R^57^ (version 4.2.1). All processing and analytical procedures for the current experiment were determined using the data from the pilot experiment without looking at the dataset (see “Overview of the experiments” section above, and the Supplementary Material).

The force–time series data were segmented into epochs of 900 ms (− 400 to 500 ms relative to tone onset) in the Rare and Roving conditions – analyses relating to these are referred to as short-interval analyses hereafter. Additionally, data from the Rare condition were segmented into longer epochs of 3400 ms (− 400 to 3000 ms relative to stimulus onset) hereafter referred to as long-interval analysis. This latter analysis was conducted for the Rare condition only, since there the onset-to-onset intervals were sufficiently long to allow the observation of potential slower force modulations^1^ without interference from consecutive tones. A reason for using two parallel analyses in the Rare condition was that longer epochs are more likely to be contaminated by artifacts, thus the short-interval segmentation provides a higher number of epochs, and thus better signal-to-noise ratio for the analyses focusing on the initial force modulations. Each epoch was baseline-corrected using the pre-stimulus interval (− 400 to 0 ms).

To reduce the influence of potential artifacts, outlier epochs were removed based on Median Absolute Deviation^58^ (MAD) of the force ranges (difference of the maximum and minimum force signal value within the epoch). To establish the rejection threshold, first the median range was calculated for all epochs (of all participants) recorded in the experiment (separately for short- and long-interval analyses). The MAD was calculated using this median range and the range-rejection threshold was then set as *‘median* + 2.5 * MAD’. Epochs with ranges exceeding the thresholds were removed from further analyses. For the short interval analyses (− 400 to 500 ms), the rejection threshold was *0.153 N.* For the long-interval analysis of the Rare condition, it was *0.266 N*. Also, to exclude epochs with “flatlines”—that is, epochs showing constant force without substantial variability (potentially caused by technical errors, or participants releasing the device), those with ranges lower than 0.01 N were also removed from the analyses. In total, 10.68% of epochs in the Rare condition and 13.37% of epochs in the Roving condition were treated as outliers and removed from the analysis. Furthermore, participants with more than 50% of epochs rejected across all the blocks in *any* experimental condition were excluded from the analyses. This resulted in the exclusion of one participant for whom 52.92% of the data were rejected across all blocks in the Roving condition.

Following the application of the rejection criteria described above, the remaining epochs of the Rare condition were averaged for each participant. In the Roving condition, average epochs were calculated separately for High and Low deviants, as well as for the final tones of the homogeneous micro-sequences. To capture deviance-induced responses in the Roving condition, differences between the epochs corresponding to deviants (i.e., the first changed tone) and the physically identical final tones of the corresponding micro-sequences were calculated—yielding two difference-waveforms corresponding to high and low tones for each participant.

### Statistical analysis

To test the presence of significant force-modulations following rare tone onsets (Rare condition), the mean force in each of the three pre-determined windows (97–117, 214–254, and 340–360 ms) for the short-interval analyses, as well as in one window (1083–1483 ms) for the long-interval analysis, was submitted to one-tailed Student’s t-tests against zero. As described in the “Overview of the experiments” section above, these window sizes and latencies were selected through the explorative analyses of the pilot data performed with the goal to maximize detection probability of force-modulation peaks resembling those reported by Novembre et al.^1^ (see Supplementary Material).

Given the Cohen’s *d* effect sizes reported in the Supplementary Material, for these analyses the sample size of 29 provided powers of 0.06, 1.00, 0.42, and 1.00 respectively, for a type I error probability of 0.05. These analyses tested whether force level in the given windows differed from that in the pre-stimulus interval (baseline). However, this approach presents an issue when applied to the difference-waveforms of the Roving condition. Although the calculation of the deviance-related responses eliminates force differences potentially emerging due to physical stimulus differences (by subtracting responses to physically identical stimuli), the baseline measure (− 400 to 0 ms intervals) involves tones that are physically different from the deviant tone, which may shift force levels in the difference waveforms by an unknown constant. To eliminate this potential confound, force differences between consecutive negative and positive peaks (calculated in pre-determined windows of 185–205, 257–297, and 403–423 ms for the low tones and 176–196, 296–336 and 386–406 ms for the high tones) were submitted to one-tailed Student’s t-tests against zero. These are referred to as peak-to-peak differences hereafter. Given the Cohen’s *d* effect sizes reported in the Supplementary Material, for these analyses, the sample size of 29 provided powers of 0.96, 1.00, 0.70, and 0.99 respectively. Because this approach provides greater statistical power to detect the presence of a modulation, these calculations were also performed for the Rare condition. Given the Cohen’s *d* effect sizes reported in the Supplementary Material, the sample size of 29 provided 1.00 power in both cases.

## Results

**Fig. 2:**
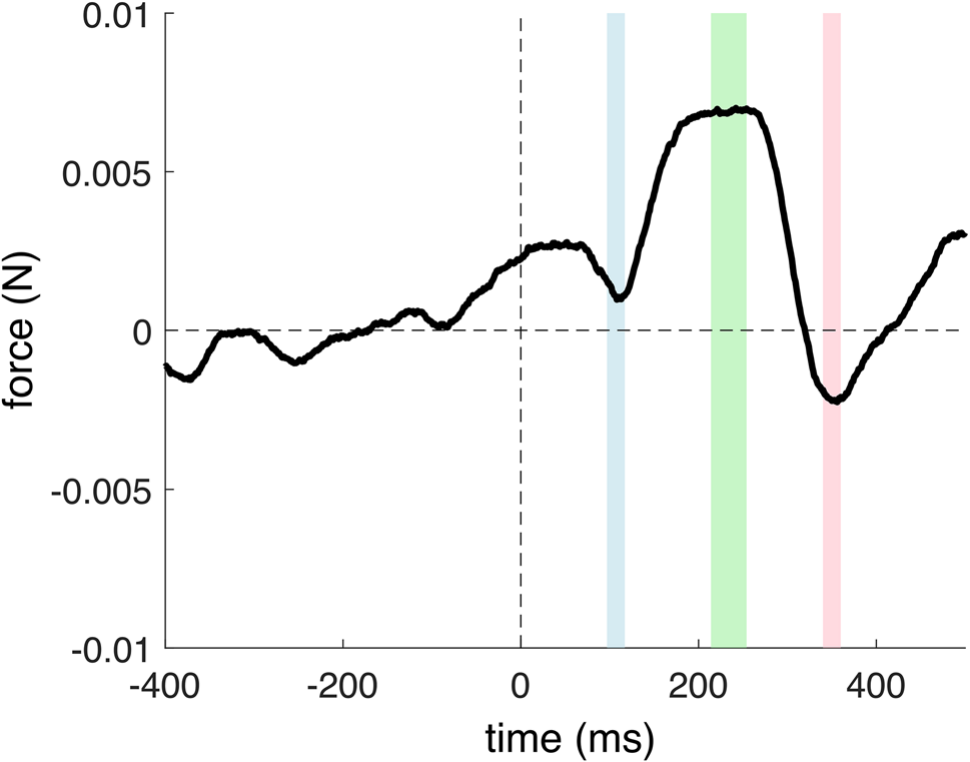
Group-mean stimulus-locked force modulation in the Rare condition in reference to rare tone onset (0 ms) in the short-interval analyses. The -400 to 0 ms pre-stimulus interval was used as a baseline. Colored intervals show the three windows-of-interest selected in the pilot experiment (see Supplementary Material).

**Fig. 3:**
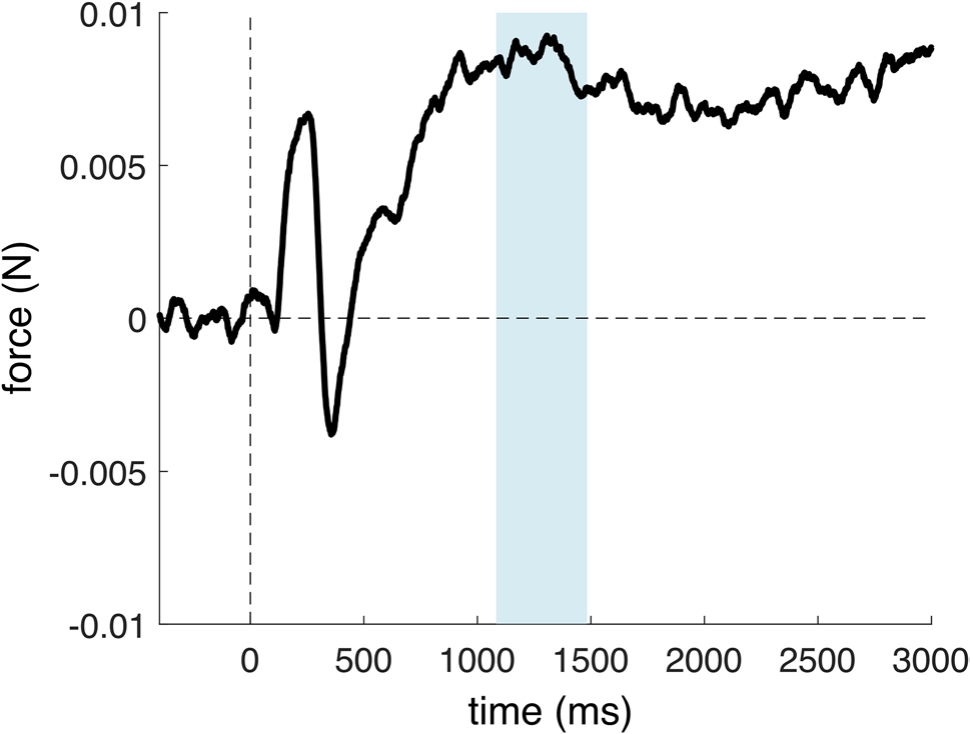
Group-mean stimulus-locked force modulation in the Rare condition in reference to rare tone onset (0 ms) in the long-interval analyses. The − 400 to 0 ms pre-stimulus interval was used as a baseline. The colored (shaded) interval shows the window-of-interest selected in the pilot experiment (see Supplementary Material).

**Fig. 4:**
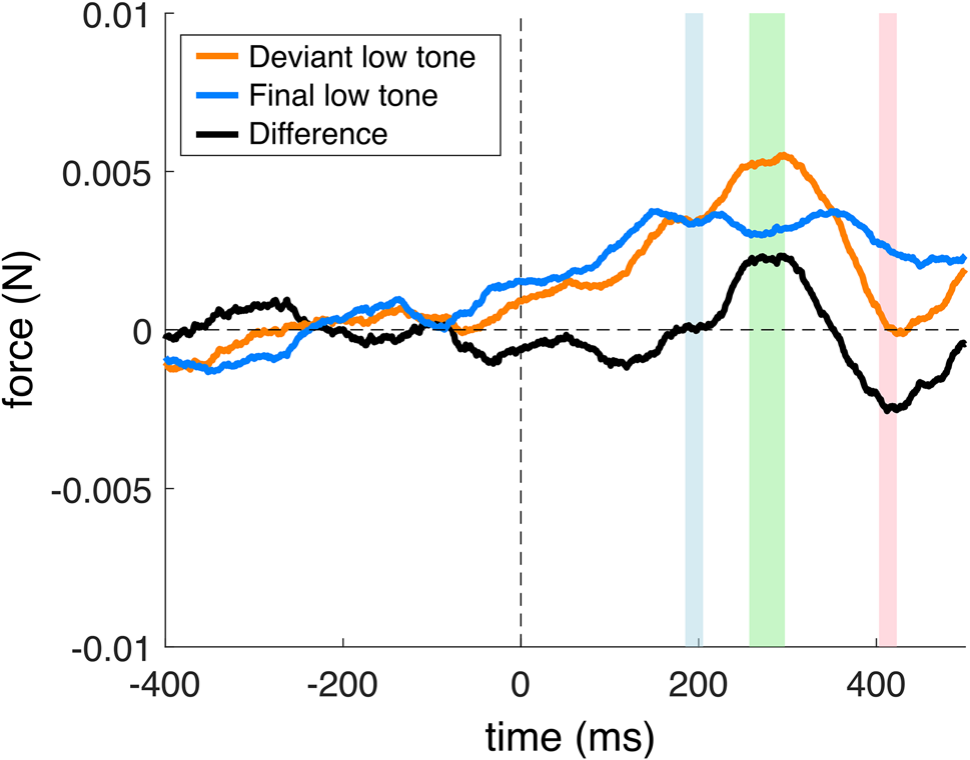
Group-mean stimulus-locked force modulation in the Roving condition in reference to the low tone onsets (0 ms). The orange line shows force modulation elicited by a low tone following a repetitive high-tone micro-sequence, that is, a *deviant*; the blue line shows the force modulation elicited by the final repetition of the low tone in a repetitive low-tone micro-sequence. The black line shows the deviant-minus-final force modulation difference. The − 400 to 0 ms pre-stimulus interval was used as a baseline. The colored intervals show the windows-of-interests selected on the basis of the pilot experiment (see Supplementary Material).

**Fig. 5:**
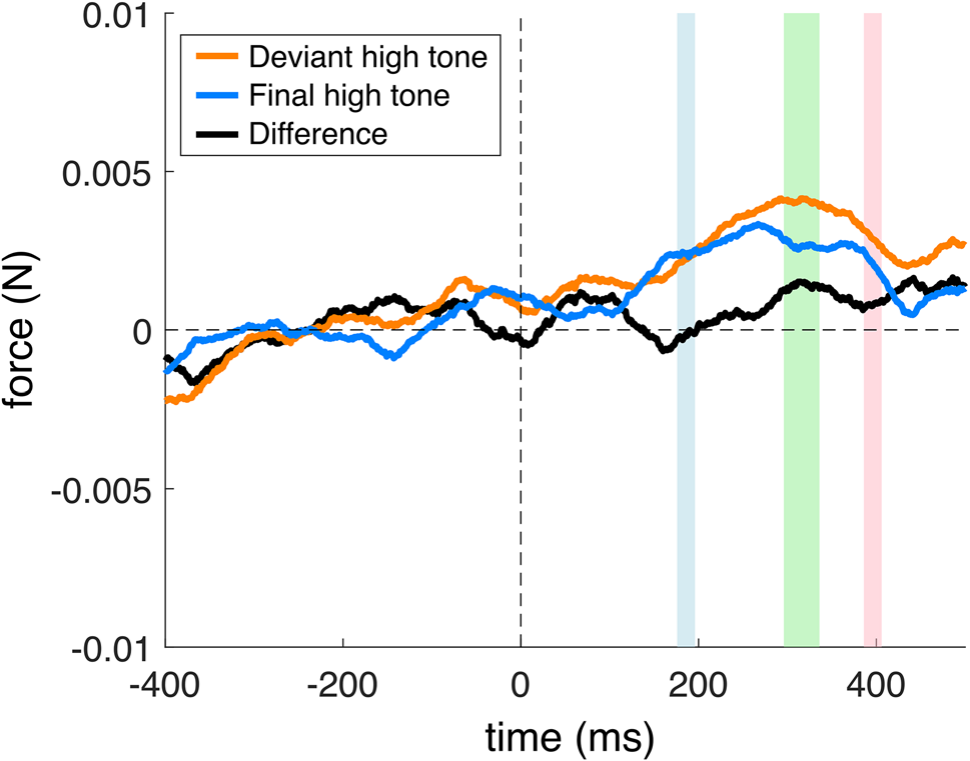
Group-mean stimulus-locked force modulation in the Roving condition in reference to the high tone onsets (0 ms). The orange line shows force modulation elicited by a high tone following a repetitive low-tone micro-sequence, that is, a *deviant*. The blue line shows the force modulation elicited by the final repetition of the high tone in a repetitive high-tone micro-sequence. The black line shows the deviant-minus-final force modulation difference. The − 400 to 0 ms pre-stimulus interval was used as a baseline. The colored intervals show the three windows-of-interests selected on the basis of the pilot experiment (see Supplementary Material).

The windows selected based on the pilot experiment matched well the actual local force peaks observed in the group-mean force time-series (Figs. 2, 3, 4 and 5). In the Rare condition, in the first window, the one-tailed* t* test did not reveal a significant negative deviation from the baseline: t(28) = 1.385, *p* = 0.912, *d* = 0.257. However, a significant increase in force was found in the second window [t(28) = 5.007, *p* < 0.001, with *d* = 0.929], as well as in the third window [t(28) = − 1.793, *p* = 0.042, *d* = 0.333]. The peak-to-peak analysis showed a significant difference between the forces in the first and second windows: t(28) = − 6.999, *p* < 0.001, *d* = 1.299, and the second and third windows: t(28) = 5.431, *p* < 0.001, *d* = 1.0085.

The long interval analysis in the 1083–1483 ms window revealed a significant force increase in comparison to the baseline: t(28) = 5.967, *p* < 0.001, *d* = 1.108 (see Fig. 3).

In the Roving condition, for the *low* tone, the comparison of force amplitudes showed significant differences between the first and second windows [t(28) = − 2.560, *p* = 0.008, *d* = 0.475] as well as between the second and third windows [t(28) = 3.657, *p* < 0.001, *d* = 0.679] (see Fig. 4).

In the Roving condition, for the *high* tone, no significant force modulations were found: there were no significant force differences between the first and second windows [t(28) = − 1.673, p = 0.053, *d* = 0.310] or between the second and third windows [t(28) = 0.712, *p* = 0.241, *d* = 0.132] (see Fig. 5).

As a post-hoc analysis, the low and high tone-related force-modulations were compared using paired, two-tailed *t*-tests. For the peak-to-peak measure between the first and second window, no significant difference was found: t(28) = 0.494, *p* = 0.625, *d* = 0.092, whereas the peak-to-peak measure between the second and third window was significantly larger for the low tone: t(28) = 3.161, *p* = 0.004, *d* = 0.587.

## Discussion

The present study demonstrated that both rare task-irrelevant sounds and unpredictable auditory changes amidst a stream of regularly presented tones modulated continuous motor output in an isometric task. The stimulus-induced motor fluctuations seemed to depend on the stimulation context, however. Indeed, when deviance was introduced as a variation within a sequence of tones, an unexpected low tone significantly impacted performance while a high tone did not. The force modulation patterns elicited by the rare tones in the Rare condition and the low deviant tones in the Roving condition were comparable: in both cases, the force exerted by participants exhibited a significant increase, followed by a sudden decrease, which emerged 50–90 ms later for low deviants. The results of the Rare condition, together with the presence of a longer lasting force increase beyond the 500 ms post stimulus onset, replicate the findings reported by Novembre and colleagues^1^. However, in contrast to their observation, we found no significant force decrease in comparison to the baseline at around 100 ms after tone onset. The results of the Roving condition extend these findings, by showing that an ongoing motor activity is not only modulated by an unpredictably presented tone after a longer period of silence, but also by a sound that deviates from the regularity of the preceding auditory stimulus sequence (when a low tone unexpectedly interrupts a sequence of high tones).

Current perspectives on sensorimotor integration mostly discuss our brain’s ability to predict upcoming events and action outcomes, prepare and eventually execute actions plans, which aids individuals to seamlessly interact with the events as they occur^59^. However, as mentioned earlier, predictability of a sudden event is not always a given—meaning, the individual may not always hold information about an upcoming event^34^. In such scenarios, a tight coupling between sensory change detection and one’s motor system would enable the individual to react in a time-sensitive manner. This is the case of the force modulations captured in our experiment, which did not rely on a memory trace of previous events or a learned motor program (as these events were either rare, or unexpected deviants among a predictable sequence of tones). The subtle muscular responses we observed were spontaneous and involuntary in nature. Recent studies on change detection emphasize that saliency-related cortical activities predict stimulus-evoked motor modulations in the sense that it drives muscle contractions, thus calling for a conceptual shift to understand it as a reactive process rather than a perceptual one^1,60^. Our results contribute to this discussion by showing that ongoing motor activity is influenced not just by the irruption of infrequent auditory events breaking periods of silences (as also shown by Novembre and colleagues^1^), but also by the occurrence of unexpected regularity violations in an auditory stream.

Although the present study used only force measurements, it is worth speculating about the neural phenomena potentially related to the observed motor effects. First, potential contributions from auditory change detection processes with characteristic neural correlates are considered, followed by some speculations on neural circuits involved in sensory-motor integration potentially playing a role in the observed motor adjustments.

In previous studies administering stimuli after longer periods without stimulation, the amplitude of the force fluctuations have been shown to correlate with changes of the vertex potential^1,38^. In the present experiment, however, force fluctuations were also found in response to unpredictably occurring frequency changes. Because these force modulations were demonstrated in change vs. repetition contrasts for sounds presented at relatively short intervals, it is clear that other—non-vertex potential-related mechanisms—brought these about. There are at least two candidate underlying mechanisms: one related to the violation of an auditory regularity reflected by MMN, and one related to the release from stimulus-specific adaptation of the auditory N1 ERP. In the present study, both mechanisms are likely to have been triggered by the unpredictably occurring frequency changes. Both of these auditory change-related ERPs have been suggested to lead to involuntary attention change^13,27^, but whereas MMN reflects the detectability of, or amount of evidence for the regularity violation^31^, stimulus-specific auditory N1 effects seem to reflect the magnitude of change^61^. The auditory N1 is also sensitive to the interstimulus interval and contributes to the vertex potential as well^6^. Although it is possible that MMN-, auditory N1-, and non-stimulus-specific change detection processes reflected by the vertex potential independently trigger somewhat similar force fluctuations, based on the present results and the characteristics of these ERPs, one may also speculate that the release from stimulus-specific adaptation is the only mechanism leading to force fluctuations. Since the release from stimulus-specific adaptation has been observed in the N120 somatosensory ERP and its magnetic counterpart^62–64^, this explanation may well accommodate previous results obtained with somatosensory stimulation^1^ as well.

Results from the Roving condition also suggest that the physical characteristics of the stimuli may influence the effect, in the sense that significant force modulations were only observed for low deviants, and a post-hoc calculation provided support for a between-condition difference in force modulation. The extent of motor responses elicited by a deviant sound may be influenced by multiple factors, like the auditory context, the task at hand etc. However, all these factors contribute to the salience of the deviant, which is what eventually drives the attentional engagement to the deviant tone. In our task, it is possible that the perceived salience of the high-frequency tone was weaker than that of low-frequency tones. There may be several possible explanations for this observation. First, the high frequency tones (4000 Hz) were used in both the Rare and Roving conditions while the low frequency tones (2996 Hz) were only used in part of the Roving condition. This may have resulted in conferring more deviance to the low tones than the high tones across the experiment, since the first may have been perceived as acoustically more distinct than the second. If so, the low tones would be more attention grabbing, and may also elicit a stronger modulation of motor responses. Second, the low tones in the Roving condition may be slightly louder than the high tones: According to equal loudness contour curves, the 4000 Hz tone should, on average, be perceived as louder than the 2996 Hz tone, all other variables being equal. Hence a run of high tones may establish a baseline in which a certain level (*L*) of acoustic energy constitutes the stimulus being repeated. If so, the low tone, yielding a smaller amount of energy (*l* < *L*) would constitute a violation of this sequence in the sense that *L* is now absent. In contrast, a high tone following a run of low tones would elicit the perception of the absence of the low tone’s energy (*L* > *l*). Though this explanation is speculative, we note that there is behavioral and electrophysiological evidence of distraction by, and responses to, omissions resembling those triggered by deviant sounds^65–72^.

A third possibility, which fits our speculation regarding the role of stimulus specific adaptation, is related to the fact that auditory excitation patterns show an “upward spread” as a function of tone frequency, which is most readily observed in masking. That is, the effect of a pure tone masker is more pronounced for frequencies higher than that of the masker (“upward spread of masking”)^73^. In terms of frequency-specific adaptation affecting the auditory N1 ERP, the high standard may cause less adaptation for the low deviant, than vice versa. Thus, the low deviant would elicit a stronger auditory N1 response than a high deviant. This possibility, however, seems at odds with previous studies, which found that frequency increments resulted in higher (or at least not lower) change-related ERP responses than decrements^61,74–78^. Some behavioral studies using frequency threshold measurements also found lower thresholds for increments than decrements^79^. In sum, this unexpected aspect of the results needs further study.

Though our study was not designed to examine the specific neural mechanisms whereby the motor system is affected by auditory processing, it is worth pointing out that past work shows the existence of specific auditory-motor neural connections (part of the dorsal auditory stream^80^, also including posterior parietal areas^81^), which, for instance, play a role in auditory sensorimotor synchronization, and in playing musical instruments^82–85^. These studies suggest that bidirectional connections between posterior auditory areas and premotor cortex allow continuous motor adjustments based on auditory feedback when producing music or maintaining motor synchrony with an external sound sequence. Importantly, the transfer of information between auditory and pre-motor cortices seems automatic^86^. In the present experiment, the goal to maintain a constant force level can be characterized as the maintenance of a dynamic balance achieved through small force adjustments compensating perceived fluctuations in tactile and proprioceptive feedback. Even though there was no contingent relationship between force application and the simultaneously presented sounds, change-related auditory processing responses may thus influence these ongoing compensatory force adjustments through the dorsal auditory stream.

On the other hand, the force modulations may have also been mediated by non-modality-specific neural circuits receiving input from multisensory and supramodal areas that process and integrate inputs from different sensory modalities, including somatosensory and proprioceptive systems. The present force fluctuations may, for example, reflect the general suppression of motor activity via fronto-basal ganglia circuits^45,87^ in response to sensory changes in auditory as well as other modalities^39^. Because the direction of attention (in terms of spatial position, inward/outward focus, towards or away from the concurrent action) has been shown to modulate motor cortical excitability^88–90^, another possibility is that the force modulations resulted from involuntary attentional shifts triggered by the detection of an auditory change^27^. These suggestions are compatible with evidence of the existence of a circuit linking the detection of unexpected changes in sensory areas (auditory, visual, or haptic) to the bilateral inferior gyri, the bilateral anterior insula and the dorso-medial prefrontal cortex, as well as the sub-thalamic nucleus^91,92^. These structures are involved in what Corbetta and Shulman^93^ referred to as the “circuit breaker”, a network at play in the stopping of one’s actions as well as in response to unexpected stimuli^87^. Of particular interest, this network receives information from the ventral auditory system^94^. Furthermore, it receives even faster input from the thalamus, connected to the pre-supplementary motor area, which is adjacent to the lateral motor cortex with a likely direct input to the sub-thalamic nucleus. According to Wessel and Aron^87^, this “putatively ancient brain system may have evolved for rapid detection of surprise, deviance, exception and prediction errors” and may constitute a “hard-wiring for a system that engages after surprising events and has the function of pausing, stopping, and clearing” (p. 273).

In the Rare condition, apart from the sudden increase and decrease in the forces within the first 500 ms post-rare stimulus onset, we also observed a positive slow wave progression beyond the short interval. It should be noted that, while applying the MAD rejection criteria (see Fig. 3.), the slow wave did not approach the baseline even after 3000 ms. In contrast, without rejecting outlier epochs, the slow wave approached baseline at around 2000 ms, similar to that reported by Novembre and colleagues^1^ (see Supplementary Material for forces plotted with no or different rejection criteria). Relaxing the outlier rejection threshold also seemed to capture the trend of the waveform approaching zero (see Fig. S6. in Supplementary Material). A speculative explanation for this could be that by rejecting epochs with larger ranges, our outlier rejection procedure may not only remove force *artifacts* unrelated to the auditory stimuli (e.g. participants changing grasp position, or accidentally releasing the device), but large-amplitude *force adjustments* occurring relatively rarely that are executed *in direct or indirect relation* to the tones. That is, our rejection criterion may have been overly conservative. However, the criteria do not seem to affect the short-interval modulations, and since the data processing protocol was established a priori using the pilot data, the criteria were not changed to capture the waveform approaching the baseline.

Our findings add to the growing evidence of a perturbation of motor processes upon the presentation of an unexpected sound. The modulations we observed are, by and large, compatible with those reported by Novembre and colleagues^1^, whereby an alternating pattern of force increase, and decrease was observed, reminiscent of the alternate bursts of activity of agonistic and antagonistic muscles in voluntary movements^95^. The data do suggest that unexpected sounds, whether rare or deviant, rapidly trigger an initial perturbation of motor processes which then ripples to produce a series of agonistic and antagonistic adjustments. The timing of the initial perturbation is difficult to determine. In our study, the earliest significant fluctuation was observed in the second time window (214–254 ms) as a force increase. While Novembre and colleagues^1^ reported a similar increase around the same time, they also picked up an earlier decrease, which is visible in our data as a local decrease but did not reach statistical significance. This discrepancy may be due to the use of different devices and the force required to operate them (which was larger in Novembre et al.’s case and hence potentially more likely to be detected statistically). Though direct comparisons are tentative due to the large differences in method and dependent variables between studies, we note that the early decrease in force reported by Novembre and colleagues^1^ is in the ballpark of the time window in which a deviant-induced reduction of corticospinal excitability was reported by Wessel and Aron^45^, and broadly similar to the temporal locus of the suppression of oculomotor movements in reading and scanning tasks^46–48^. Our study suggests that such perturbation of the motor system is not specific to deviant sounds^45,48^ or specific to rare sounds^1^, but is observed with both. It is compatible with the notion of action uncertainty in the face of unexpected events whereby the organism interrupts ongoing actions to take stock of changes in the immediate environment and modify, if necessary, its behavior. Change detection forms an essential part of our arsenal to deal with our environment and occurs incredibly fast, with evidence showing a response at the level of the brain stem as early as 20–40 ms from the sound’s onset^96–98^. The speed of this response, together with evidence of the role of the brainstem in eye movements^99,100^ and its connection to motor areas^101^ fit with the notion that unexpected events trigger a lightning fast and automatic detection of a prediction error, which triggers the inhibition of ongoing motor activities, an orienting response toward the unexpected stimulus, and an adjustment of one’s actions if necessary^8^.

### Supplementary Information

Supplementary Information.

## Data Availability

All data, stimulation and analysis scripts are available at https://osf.io/6b2vs/.
