# Supplementary material for: Involuntary motor responses are elicited both by rare sounds and rare pitch changes
Source: Sci Rep. 2024 Aug 30;14:20235. doi: 10.1038/s41598-024-70776-x (PMC11364668; doi:10.1038/s41598-024-70776-x)
Supplement: Supplementary file 1 — Supplementary Information. [file 41598_2024_70776_MOESM1_ESM.docx]

# **Supplementary Material**

# Involuntary motor responses are elicited both by rare sounds and rare pitch changes

Simily Sabu, Fabrice B. R. Parmentier, János Horváth

# **Pilot Experiment**

## **Materials and Methods**

***Participants****.* A total of 20 healthy human participants (19 females, mean ± SD age = 22.1 ± 1.889 years, range 19-26 years, 4 left-handed) participated in the experiment. One participant’s data was excluded based on the outlier rejections (see below); thus 19 participants were included in the final analysis (18 females, mean ± SD age = 21.19 ± 1.87 years, range 19-26 years, 4 left-handed). All participants gave their written informed consent prior to the experiment. The project was approved by the United Ethical Review Committee for Research in Psychology (Hungary).

***Apparatus, stimuli, and procedure***. The pilot study followed the same protocol as the main experiment, except for the familiarization phase and the beginnings of the experimental blocks, during which continuous visual feedback was provided about the force produced by participants. This feedback took the form of a level represented by the vertical position of a dot on the screen, which traced a line on the screen (as if the screen rolled horizontally with a constant speed, see S1 Fig. 1). Parallel horizontal lines were also visible, indicating the target force range. In the experimental blocks, in which participants maintained a steady force-level within the pre-set range, the experimenter started the stimulation and in the absence of visual feedback.

***Data processing.*** Data processing was the same as in the main experiment, except for baselining: To exclude the interval contaminated by artefacts just before tone onsets, the -400 to -100 ms interval was used for baseline calculation (instead of -400 to 0 ms).

The criteria used for outlier rejection was based on MAD as described in the main text. The rejection threshold for the pilot data (N = 20) was 0.130 N for the short-interval analyses, and 0.232 N for the long interval analysis of the Rare condition. This led to the removal of 12.35% of the Rare condition epochs and 14.83% of the epochs from the Roving condition. Finally, one participant was excluded from due to high rejection rate - 50.25% in the Roving condition).


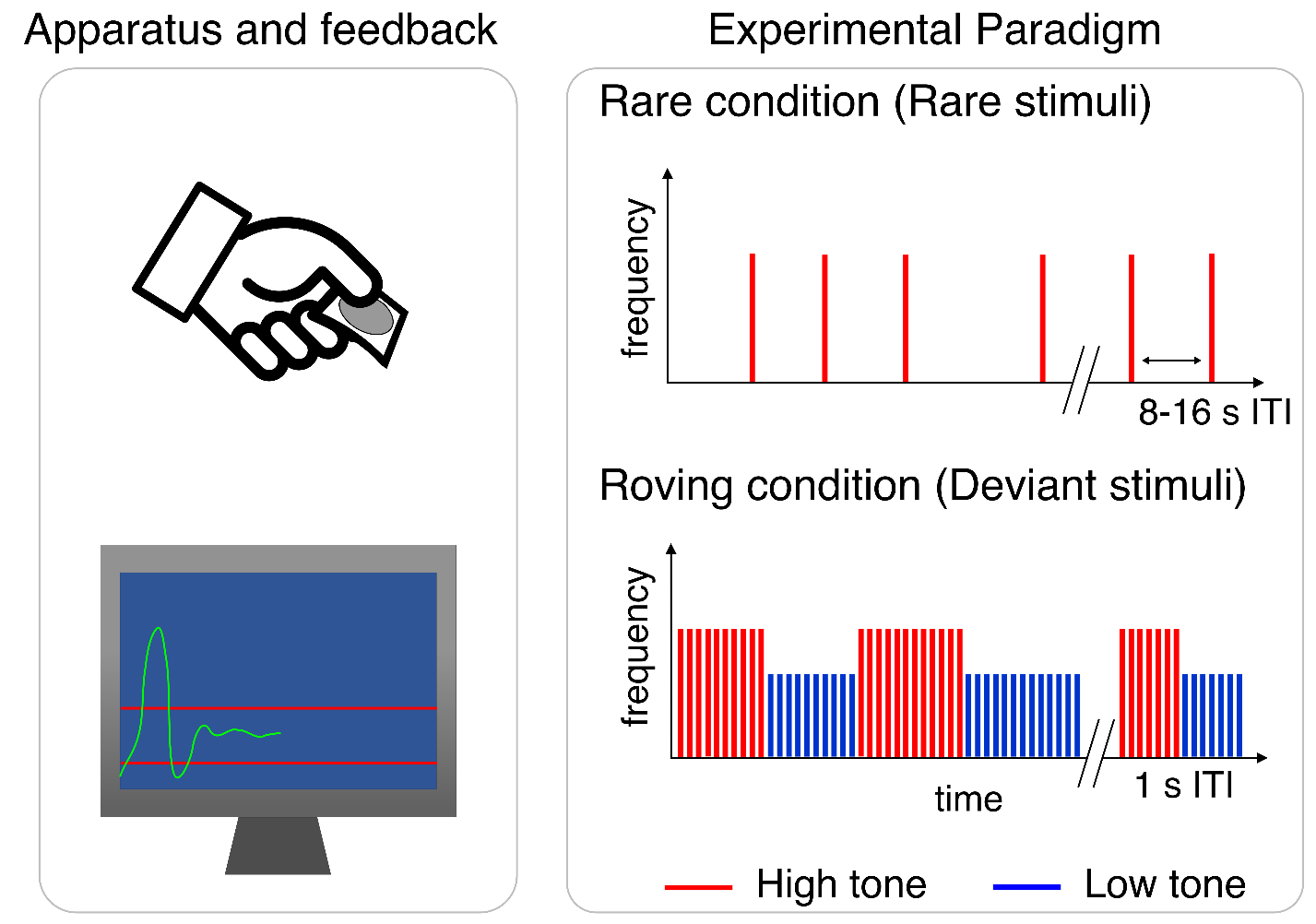


**Figure S1. Experimental method and paradigm**. Top left: participants were required to produce a 1-2 N range force by pinching a Force Sensitive Resistor between their thumb and index finger maintaining a thumb-above grip position. Bottom left: continuous visual feedback was presented during the familiarization phase indicating to participants the force level being produced (green waveform) along with the target force range, 1-2 N (red horizontal lines). Right panel: depicting the experimental paradigm is the same as described in the main manuscript.

***Data exploration/analysis.*** To explore the optimal parameters to detect a force deviation from the baseline, mean force was calculated in sliding windows of 5, 10, 20, 40, 80 ms, moved in 1 ms steps along the time series. Individual average forces were submitted to Student’s one-sample, one-tailed t-tests against zero for each window. Window size and temporal position was selected to maximize the t-value for each peak, but for peak selection we also considered the similarity of the present group-mean force time-series results to those reported by Novembre et al. (2018), and whether window size was commensurate with force peak width (longer windows may “smear out” peaks, whereas shorter windows may be not optimal to detect a peak, should a temporal shift occur in the main experiment).

**Results**

In the Rare condition, a 20 ms window centered at 107 ms captured well a local negative force peak, although no statistically significant effect was found: *t*(18) = -0.070, p =.472, *d* = 0.016 (see S1 Fig. 2). The following positive peak was well captured by a 40 ms window centered at 234 ms: *t*(18) = 5.114, p < .001, *d* = 1.17. A second negative peak was adequately captured by a 20 ms window centered at 350 ms, although it did not reach statistical significance: t(18) = -1.203, p = .122, *d* = 0.275. The latency and shape of these waveforms were similar to those reported by Novembre et al- (2018).


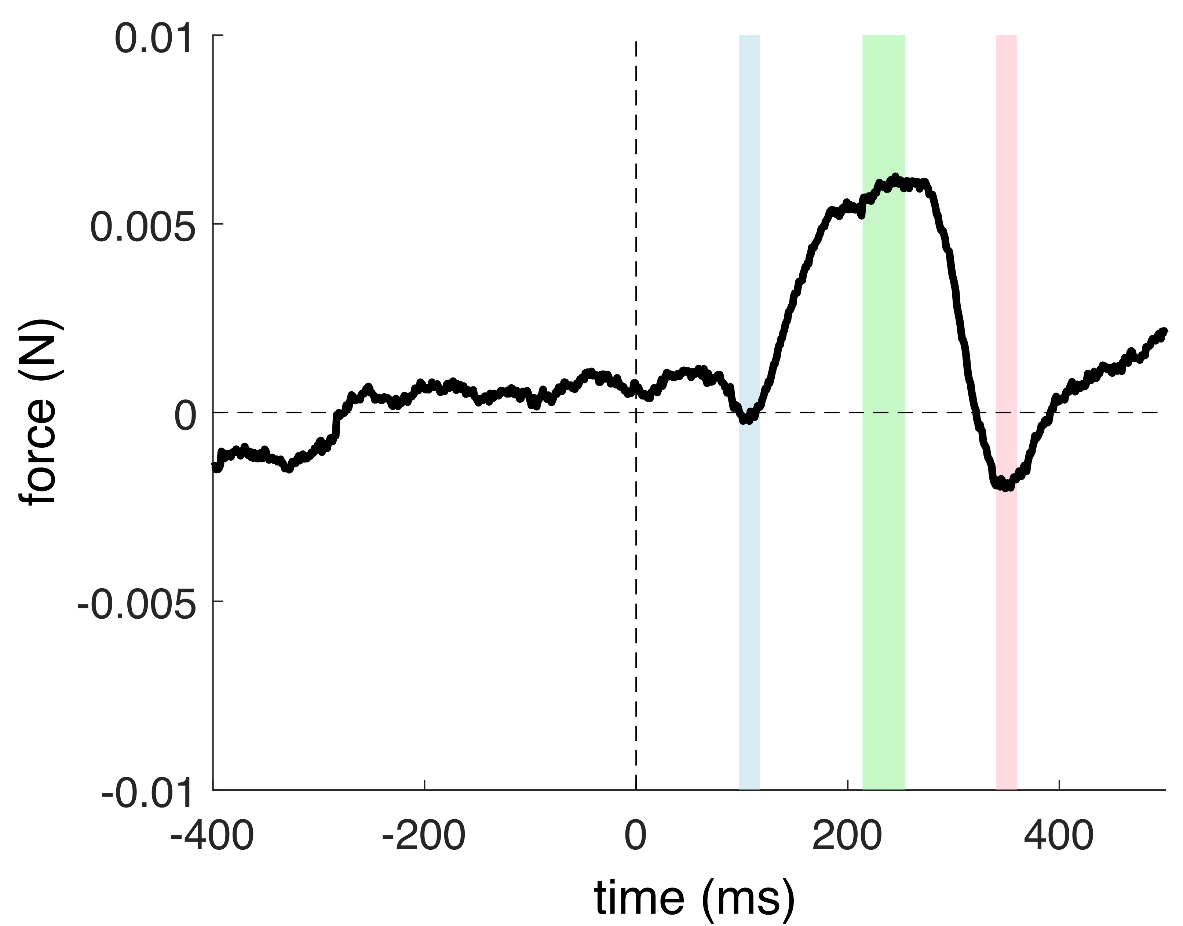


**Figure S2**. **Group-mean stimulus-locked force modulation in the Rare condition in reference to rare tone onset (0 ms) in the short-interval analyses.** The -400 to -100 ms pre-stimulus interval was used as a baseline. Colored intervals show the three time windows established by detecting the peaks corresponding to the maximum *t*-value observed from subjecting the average force to a one-sample t test against zero. A local negative peak was captured by a 20 ms window centered at 107 ms, followed by a positive peak at 234 ms with a 40 ms window width, and another negative peak with a 20 ms window centered at 350 ms. Thus, windows of interest for short interval analysis in the Rare condition for the main experiment were established as 97-117, 214-254, and 340-360 ms.

As described in the main text, the probability to detect the presence of a force-modulation can be maximized by comparing consecutive negative and positive force peaks (instead of comparing these to the baseline). One-tailed paired *t-*tests showed significant differences between the first and the second peak, *t*(18) = -7.240, p < .001, *d* = 1.66, as well as the second and the third peak, *t*(18) = 4.444, p < .001, *d* = 1.019.

The slow-wave progression in the long-interval epoch was also analysed. We found that a 400 ms window centered at 1283 ms captured the slow positive wave well: The one-tailed *t*-test against zero showed a significant force increase: *t*(18) = 3.872, p<0.001, d = 0.88 (see S1 Fig. 3).

**
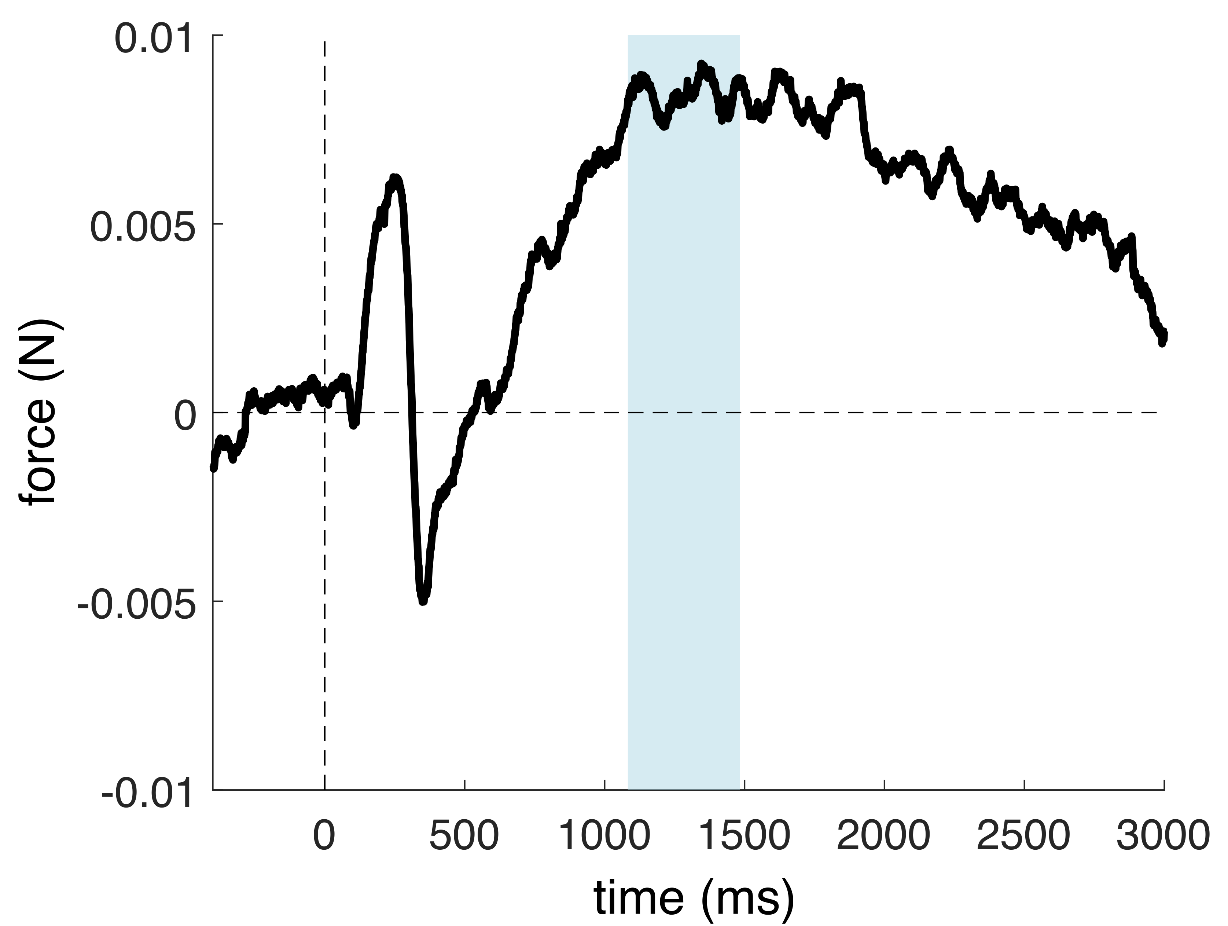
**

**Figure S3. Group-mean stimulus-locked force modulation in the Rare condition in reference to rare tone onset (0 ms) in the long-interval analyses.** The -400 to -100 ms pre-stimulus interval was used as a baseline. The colored interval shows a 400 ms window capturing the positive peak at 1283 ms of the slow wave progression. Thus, the window of interest for long interval analysis in the Rare condition for the main experiment was established as 1083-1483 ms.

In the Roving condition, a one-tailed *t-tests* against zero were conducted on the difference waveform calculated as the difference in force signals corresponding to the deviant tone and the last instance of the same tone in a homogeneous micro-sequence (see main text for details). These analyses were ran seperately for low and high frequencies so as to avoid potential confounds due to physical tone differences. In the selection of the intervals, similarity of the three-phase (negative-positive-negative) waveform to that reported by Novembre et al. (2018) played a substantial role.

For the low tone, a 20 ms window centered at 195 ms was selected to capture the first negative peak, even though the average force within the window did not significantly differ from the baseline: *t*(18) = -0.783, p = .222, *d* = 0.179 (see S1 Fig. 4). A 40 ms window centered at 277 ms was selected to capture the following positive peak, even though the t-test showed no significant difference from the baseline, *t*(18) = 1.130, p = .137, *d* = 0.25 . The final negative peak was well captured by a 20 ms window centered at 413 ms: *t*(18) = -3.718, p < .001, *d* = 0.729. Comparisons of the first and second peak showed significant force difference between the first window and the second: *t*(18) = -2.7953, p = .006, *d* = 0.641, as well as the second and the third window, *t*(18) = 4.581, p < .001, *d* = 1.051.


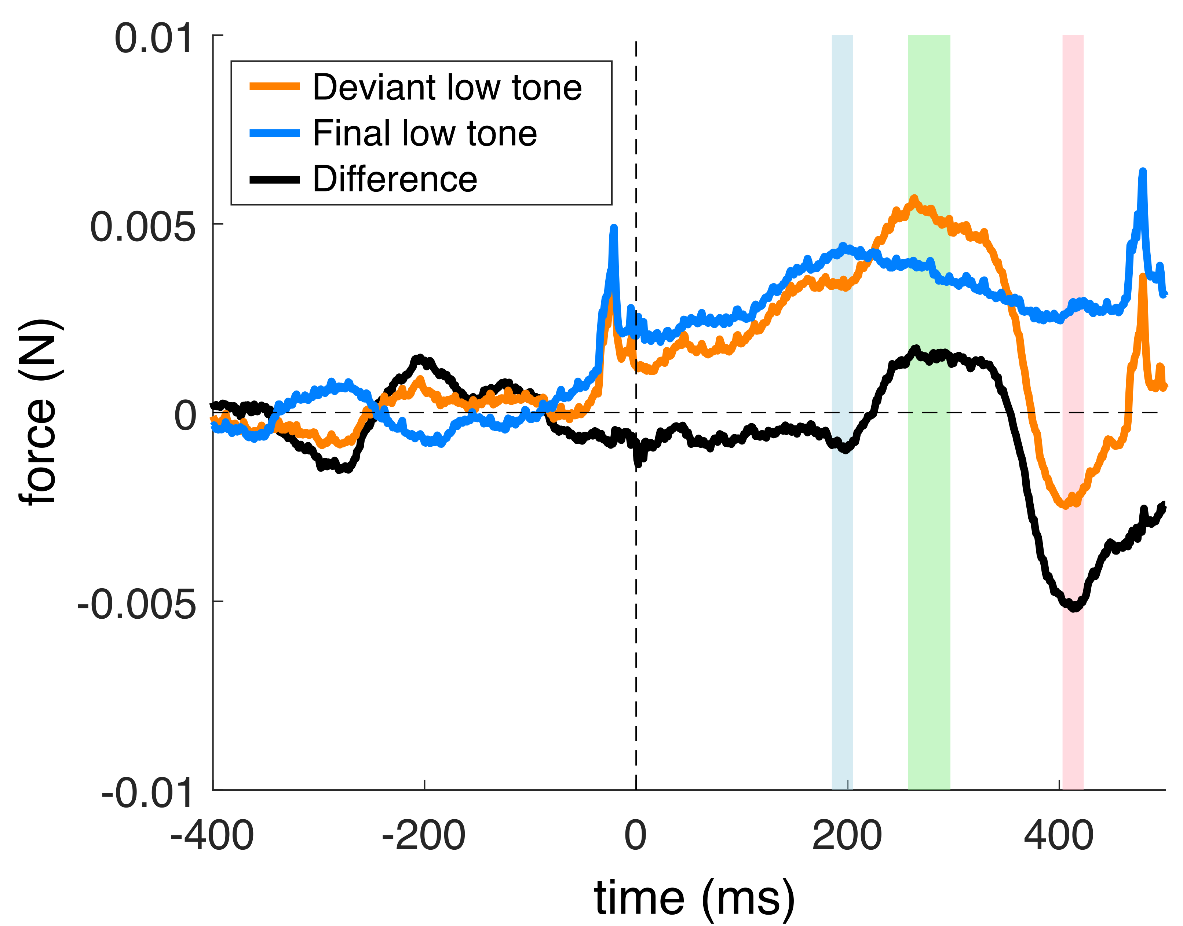


**Figure S4. Group-mean stimulus-locked force modulation in the Roving condition in reference to the low tone onsets (0 ms).** The orange line shows force modulation elicited by a low tone following a repetitive high-tone micro-sequence, that is, a *deviant*. The blue line shows the force modulation elicited by the final repetition of the low tone in a repetitive low-tone micro-sequence. The black line shows the deviant-minus-final force modulation difference. The -400 to -100 ms pre-stimulus interval was used as a baseline. The artifact is visible as a sharp positive peak shortly preceding the tone onset (and shortly before 500 ms). Colored intervals show the three time-windows established by detecting the peaks corresponding to the maximum *t*-value against zero: a negative force peak captured by a 20 ms window centered around 195 ms, a positive peak captured by a 40 ms window centered at 277 ms, and another negative peak captured by a 20 ms window centered at 413 ms. Thus, windows of interest for short interval analyses in the Roving condition with low tones were established as 185-205, 257-297, and 403-423 ms.

For the high tone, a 20 ms window centered at 186 ms was selected, even though no significant difference from the baseline was found: *t*(18) = 0.788, p = .780, *d* = 0.180 (see S1 Fig. 5). A 40 ms window centered at 316 ms was selected for the following positive peak and was found to be significantly different from the baseline: *t*(18) = 2.094, p = .025, *d* = 0.483. Finally, a 20 ms window centered at 396 ms was selected to capture the final negativity, even though no significant difference was found: *t*(18) = -0.168, p = .434, *d* = 0.038. Comparisons of the (negative) first and (positive) second peaks showed a significant difference: *t*(18) = -1.811, p = .043, *d* = 0.415, as well as the comparison of the second (positive) and third (negative) peak: *t*(18) = 3.308, p = .002, *d* = 0.759.


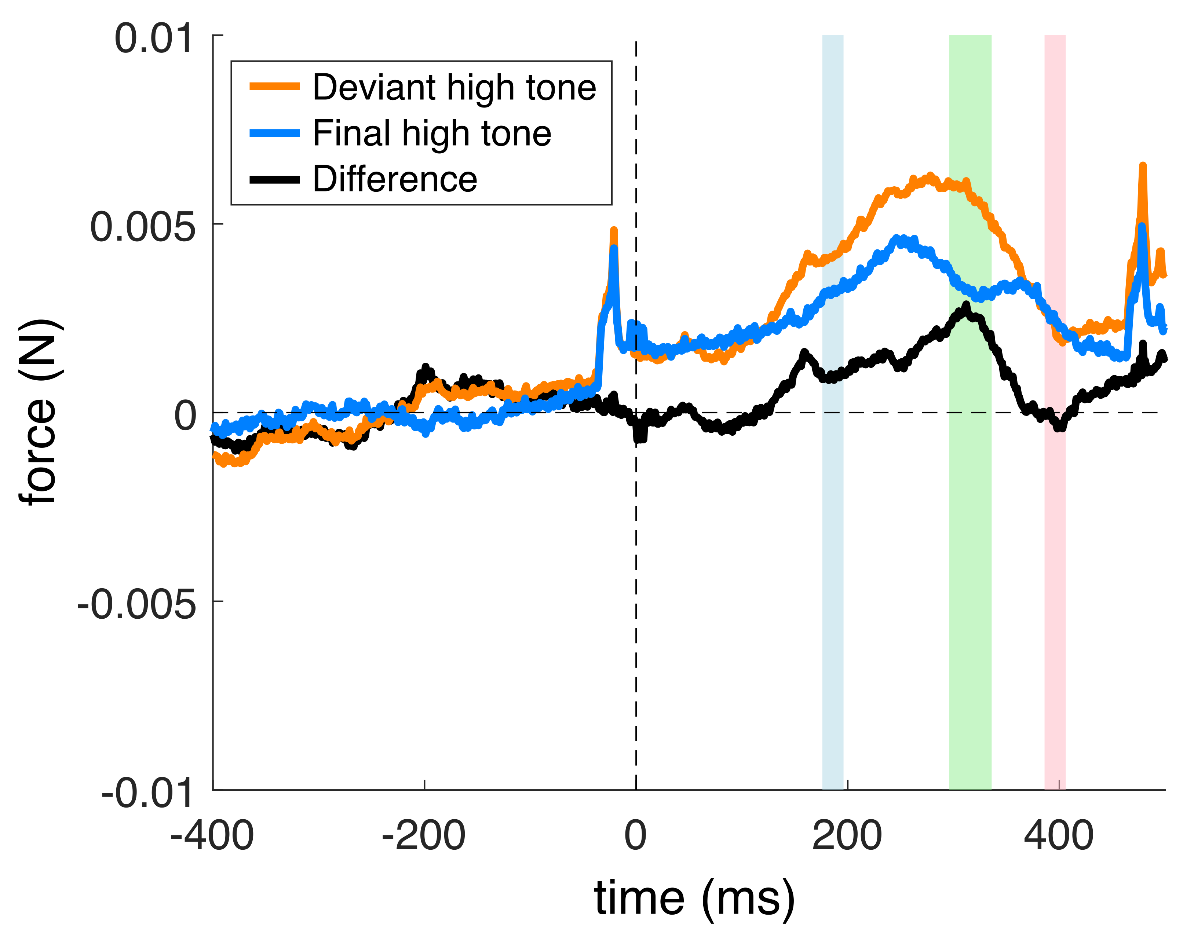


**Figure S5. Group-mean stimulus-locked force modulation in the Roving condition in reference to the high tone onsets (0 ms).** The orange line shows force modulation elicited by a high tone following a repetitive low-tone micro-sequence, that is, a *deviant*. The blue line shows the force modulation elicited by the final repetition of the high tone in a repetitive high-tone micro-sequence. The black line shows the deviant-minus-final force modulation difference. The -400 to -100 ms pre-stimulus interval was used as a baseline. The artifact is visible as a sharp positive peak shortly preceding the tone onset (and shortly before 500 ms). Colored intervals show the three time-windows established by detecting the peaks corresponding to the maximum *t*-value against zero: a negative force peak captured by a 20 ms window centered around 186 ms, a positive peak captured by a 40 ms window centered at 316 ms, and another negative peak captured by a 20 ms window centered at 396 ms. Thus, the windows of interest for short interval analyses in the Roving condition with high tones were established as 176-196, 296-336 and 386-406 ms.

**Force modulation in the Rare condition with different rejection criteria applied**


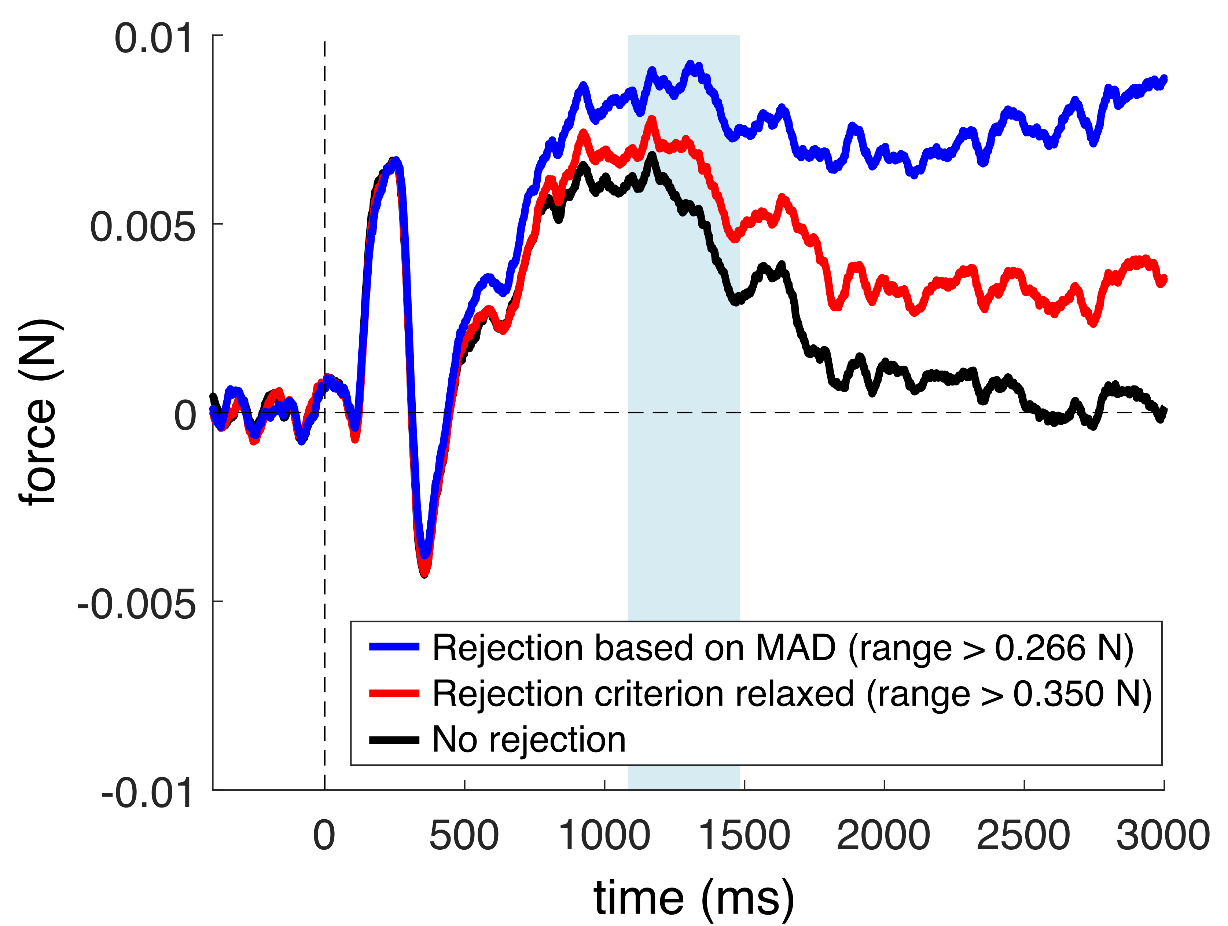


**Figure S6.** Group-mean stimulus-locked force modulation in the Rare condition in reference to rare tone onset (0 ms) in the long-interval analyses. The -400 to 0 ms pre-stimulus interval was used as a baseline. The three lines depict force modulations with no or different outlier rejection criteria applied. The colored interval shows the window of interest selected in the pilot experiment.
